# Same Defocus, Different Vision: The Role of Astigmatism Type in Pseudophakic Visual Acuity Loss

**DOI:** 10.1007/s44402-026-00087-3

**Published:** 2026-04-30

**Authors:** Johannes Weisensee, Otmar Michael Ringhofer, Achim Langenbucher, Nóra Szentmáry

**Affiliations:** 1 Department of Experimental Ophthalmology, Saarland University, Homburg, Germany; 2 Chiemsee Augentagesklinik, Prien am Chiemsee, Germany; 3 Department of Ophthalmology, Semmelweis University, Budapest, Hungary

**Keywords:** Defocus equivalent, Prediction model, Pseudophakia, Residual astigmatism, Visual acuity.

## Abstract

**Purpose:**

To investigate how the configuration of residual astigmatism, defined by the location of Sturm’s conoid, affects visual acuity loss in pseudophakic eyes, and to compare scalar and vector-based defocus equivalents as predictors.

**Methods:**

This retrospective study included 75 pseudophakic eyes post cataract surgery with aspheric or toric monofocal intraocular lenses. Manifest refraction was measured 4–8 weeks postoperatively. Eyes with residual refractive cylinder between 0.50 and 1.25 D and best corrected distance visual acuity (CDVA) ≤ 0.20 logMAR were included. Visual acuity loss (VAL) was defined as uncorrected distance visual acuity (UDVA) minus CDVA. The defocus equivalent (DEQ) was calculated using two methods. DEQ_vec_ was derived as the Euclidean norm of the power-vector components, whereas DEQ_abs_ was calculated using the standard scalar formula as the sum of the absolute spherical equivalent and half the absolute cylinder. Refractions were classified as simple, mixed or compound astigmatism. Proportional regression slopes were determined for visual acuity loss against DEQ_abs_ and DEQ_vec_.

**Results:**

VAL showed a robust linear relationship with DEQ_abs_, with a slope of 0.195 logMAR per dioptre (*R*² = 0.80). Stratified slopes were 0.12, 0.22 and 0.24 logMAR/D for simple (*n* = 50), mixed (*n* = 7) and compound astigmatism (*n* = 18), respectively, indicating that simple astigmatism produced approximately half the VAL per dioptre of compound astigmatism. DEQ_vec_ yielded a slightly steeper overall slope (0.250 logMAR/D, *R*² = 0.83) but did not change the ranking of astigmatism types.

**Conclusion:**

The impact on UDVA depends not only on DEQ, but also on residual astigmatism type. DEQ_vec_ yielded marginally higher *R*² than DEQ_abs_ but did not change the ranking of astigmatism types. In eyes with moderate residual cylinder, compound astigmatism caused the largest and simple astigmatism the smallest VAL per dioptre of defocus.

Key Points

- Distance visual acuity loss in pseudophakic eyes rises almost linearly with defocus equivalent.
- Compound astigmatism shows about twice the visual acuity loss of simple astigmatism, with mixed astigmatism in-between.
- Simple myopic astigmatism was associated with less functional impact than a mixed configuration in eyes corrected with non-toric intraocular lenses; prospective confirmation is needed.

## Introduction

Visual acuity is one of the most important endpoints used to evaluate outcomes after cataract surgery. Modern small-incision phacoemulsification with aspheric intraocular lenses (IOLs) routinely achieves excellent corrected distance visual acuity (CDVA); however, everyday visual function strongly depends on the amount and type of the residual refractive error. Even small residual refractive errors can lead to measurable loss of uncorrected visual acuity, particularly when residual astigmatism is present. For this reason, refractive predictability has become a central quality metric, and many studies have focused on the proportion of eyes within ±0.50 D or ±1.00 D of the target refraction [1–4].

Beyond simple spherical equivalent (SEQ) and cylinder magnitude, several optical models have been proposed to describe how refractive errors degrade visual acuity. Early work by Raasch and others treated the combined effect of sphere and cylinder as a root-mean-square (RMS) blur term, which can be related to visual acuity loss (VAL) [5, 6]. Later approaches, such as the defocus equivalent (DEQ), aimed to summarise the total amount of low-order refractive blur in a single dioptric quantity, making it more convenient for clinical use [7, 8]. Holladay and co-workers proposed a scalar DEQ that combines spherical and astigmatic components into a single number, while Blendowske introduced a simplified linear formulation that closely approximates the RMS blur over a wide range of refractions [6, 9]. In a recent study, we showed in a pseudophakic post-cataract population that the absolute defocus equivalent (DEQ_abs_) is a robust scalar predictor of VAL, with an almost proportional relationship between DEQ_abs_ and loss of visual acuity at different testing distances [10].

However, all of these scalar approaches deliberately reduce residual refraction to a single magnitude of blur. They do not account for the geometrical configuration of astigmatic errors in dioptric space. In astigmatism, two principal focal lines form Sturm’s conoid, with the circle of least confusion (COLC) located between them. Depending on the relative position of these planes, one can distinguish myopic and hyperopic simple astigmatism, compound myopic or hyperopic astigmatism and mixed astigmatism. From an optical point of view, one might assume that the same scalar amount of blur (e.g., the same DEQ_abs_) may have different functional consequences if both focal lines lie in front of or behind the retina, or if the retina is located between them. However, empirical data on how the type of astigmatism and the position of Sturm’s conoid modulate VAL in pseudophakic eyes are limited.

This question is closely linked to the way IOL power calculation is currently implemented in clinical practice. Contemporary formulas, as provided in common biometers, typically report the predicted postoperative refraction as a target SEQ. In astigmatic eyes without toric IOLs, aiming for an emmetropic SEQ almost inevitably leads to a mixed astigmatism configuration: one focal line is located in front of and the other behind the retina, with the COLC on or near the retinal plane. This configuration appears ‘balanced’ in terms of defocus, but it is self-evident that it does not minimise VAL for a given amount of residual blur. From a clinical perspective, an alternative strategy would be to accept a slight myopic bias and aim for a simple myopic configuration, where one focal line is located on or near the retina and the other in front of it.

Such a configuration might lead to less VAL per dioptre of DEQ compared with compound astigmatism, where both focal lines are displaced from the retina. Whether simple astigmatism also outperforms mixed astigmatism, in which the COLC lies near the retinal plane, is less obvious and was a specific question of this study.

To overcome the limitations of scalar metrics, vectorial formulations—specifically power vectors (*M, J*_0_*, J*_45_)—retain directional information by treating residual refraction as a coordinate in three-dimensional dioptric space. Based on this representation, the vector-derived defocus equivalent (DEQ_vec_) was calculated as the Euclidean norm of these components, representing the total distance from emmetropia (identical to the ‘blur strength’ *B* defined by Thibos et al. [11]). In predominantly astigmatic refractions, such a vectorial description might yield a higher *R*^2^ value than a purely scalar measure, particularly when the configuration of Sturm’s conoid is taken into account.

The purpose of the present study was to investigate how the type of residual astigmatism influences VAL in pseudophakic eyes with a moderate amount of astigmatism. The analysis was restricted to eyes with a refractive cylinder between 0.50 and 1.25 D; such astigmatism is expected to have a clinically relevant impact on visual acuity while excluding cases of high residual astigmatism. Building on our previous work on the DEQ, the performance of scalar DEQ_abs_ and its vectorial counterpart DEQ_vec_ was compared in predicting VAL at a distance. Furthermore, analysis sought to indicate whether the relationship between DEQ and VAL is systematically modulated by the type of astigmatism and the position of Sturm’s conoid, with particular focus on the potential advantage of myopic simple configurations over the ‘default’ mixed astigmatism implied by targeting an emmetropic SEQ.

## Methods

### Study Design and Population

This retrospective observational study included 75 pseudophakic eyes of 60 patients following routine cataract surgery at a single clinical centre. The study adhered to the tenets of the Declaration of Helsinki. The Ethics Committee of the Saarland Medical Association (Ärztekammer des Saarlandes) granted approval and waived the requirement for informed consent (approval No. 157/21). Eligible eyes had undergone uneventful small-incision phacoemulsification with implantation of either a non-toric aspheric monofocal IOL (CT Asphina 409MP, Carl Zeiss Meditec, zeiss.com) or a bitoric aspheric monofocal IOL (AT Torbi 719MP, Carl Zeiss Meditec, zeiss.com) from the same platform. In order to maximise the sample size and cover the refractive range necessary for regression analysis, both eyes of 15 patients were included, while 45 patients contributed only one eligible eye. Potential inter-eye correlations were addressed using patient-level cluster bootstrapping for confidence interval (CI) estimation.

Inclusion criteria for the present project were:

- uneventful cataract surgery with in-the-bag IOL implantation,
- availability of a standard postoperative examination 4–8 weeks after surgery,
- postoperative CDVA of 0.20 logMAR or better,
- availability of reliable postoperative subjective spherocylindrical refraction (following standard clinical protocols, standardised to minus-cylinder notation) and corresponding distance visual acuity,
- postoperative refractive cylinder magnitude between 0.50 and 1.25 D (inclusive),
- magnitude of residual defocus equivalent (DEQ_abs_) ≥ 0.25 D, to ensure clinical relevance and a clear separation from emmetropia.

Eyes with a history of ocular surgery, corneal ectatic disease such as keratoconus or pellucid marginal degeneration, corneal scars potentially affecting visual function, uncontrolled intraocular pressure elevation, clinically relevant macular or other retinal pathology affecting visual prognosis or postoperative complications were excluded. Eyes with signs of temporary corneal oedema were excluded by comparing pre- and postoperative pachymetry or anterior segment findings in the clinical record.

The restriction to a moderate range of residual refractive cylinder (0.50–1.25 D) was chosen to reflect the clinically relevant interval for ‘residual cylinder’ in modern cataract surgery and to avoid extreme astigmatic configurations with very large DEQ values that are less typical as a planned target state.

### Surgical Technique and IOL Calculation

All surgeries were performed by the same experienced surgeon (O.M.R.) using a standardised micro-incision technique. A clear-corneal main incision of 2.2 mm was placed temporally at the peripheral cornea, complemented by two paracenteses of approximately 1.0 mm. Phacoemulsification was carried out using a chop technique with a modern phaco system (QUATERA 700, Carl Zeiss Meditec, zeiss.com), followed by implantation of an intraocular lens into the capsular bag (CT Asphina 409MP or AT Torbi 719MP, Carl Zeiss Meditec, zeiss.com).

Biometric measurements for IOL power calculation were obtained preoperatively with the IOLMaster 700 (Carl Zeiss Meditec, zeiss.com) using total keratometry. The dioptric power of the non-toric IOLs was calculated with Barrett U2TK, the toric IOLs were calculated with the ZCalc formula (Carl Zeiss Meditec, zeiss.com) according to the standard procedures at the centre. The present analysis focused solely on the achieved postoperative manifest refraction and visual acuity; details of the IOL calculation strategy were not considered in this study.

### Refraction, Visual Acuity and Astigmatism Classification

Postoperative examinations were carried out 4–8 weeks after surgery. Subjective refraction and visual acuity were assessed by experienced optometrists using a monocular refraction protocol. Number optotypes were presented on an LCD chart screen (ULC-900 RG, Unicos, unicos.com). The refractive sphere was refined in 0.25 D steps following the maximum-plus principle, and cylinder power and axis were refined with a ±0.25 D Jackson cross-cylinder. Chart systems were calibrated to a test distance of 6.0 m to ensure constant angular subtense of the optotypes according to International Standards Organization  (ISO) 8596 [12]. Subjective refraction was performed at this test distance to avoid vergence errors. Ambient lighting conditions were in accordance with ISO standards [13].

Manifest refraction was determined in minus-cylinder notation as sphere (SPH), cylinder (CYL) and cylinder axis (AX). For each eye, uncorrected distance visual acuity (UDVA) and best-CDVA measured with full spherocylindrical correction in the trial frame were documented. VAL at distance is referred to as the difference between uncorrected and best corrected acuity in logMAR units, so that positive values indicate a loss of acuity due to residual refractive error.

### Classification According to Sturm’s Conoid

To analyse the interaction between VAL and the position of the focal lines relative to the retina (Sturm’s conoid), postoperative refractions were classified into astigmatic types based on the two principal meridians. In minus-cylinder notation, the principal powers were calculated as:

$${F}_{1}={SPH}{{;F}}_{2}={SPH}+{CYL}$$

The retinal plane was referenced to 0.00 D (distance emmetropia). Using a tolerance band of ±0.25 D around the retinal plane, refractions were assigned to the following categories:

- Simple myopic astigmatism: *F*_1_ within ±0.25 D (the retinal plane); *F*_2_ < −0.25 D.
- Simple hyperopic astigmatism: *F*_2_ within ±0.25 D (the retinal plane); *F*_1_ > +0.25 D.
- Compound myopic astigmatism: *F*_1_ and *F*_2_ < −0.25 D.
- Compound hyperopic astigmatism: *F*_1_ and *F*_2_ > +0.25 D.
- Mixed astigmatism: *F*_2_ < −0.25 D and *F*_1_ > +0.25 D.

For the main analysis, compound myopic and compound hyperopic cases were pooled into a compound group, while the two simple astigmatism groups were pooled into simple astigmatism if needed. Additionally, simple myopic and simple hyperopic astigmatism were analysed separately to evaluate whether the direction of the associated spherical error influenced VAL. Particular emphasis was placed on simple myopic astigmatism versus compound/mixed configurations, because in simple myopic cases, one focal line lies on or close to the retina, whereas in mixed astigmatism, the retina lies between the two focal lines and in compound astigmatism, both focal lines lie on the same side of the retina.

For the present analysis, the orientation of the cylinder axis was not considered, as the relationship between axis and visual acuity is strongly dependent on optotype layout and was not a primary focus of the study.

### Defocus Equivalents and Outcome Parameters

Based on SPH, CYL and AX, predictors of defocus were calculated for each eye:

The SEQ was calculated using the standard formula:

$${SEQ}=\,{SPH}+(1/2\,C)$$

#### Defocus Equivalent (Based on Holladay)

To quantify the scalar amount of blur created by the combination of spherical and cylindrical errors, the absolute defocus equivalent (DEQ_abs_) was calculated. As established in our previous work and following the concept of equivalent blur described by Holladay et al., this metric serves as a practical predictor of VAL [9, 10]:

$${DEQ}={ABS}\left({SPH}+1/2{CYL}\right)+{ABS}\left(1/2{CYL}\right)$$

#### Vector-Dioptric Distance (Based on Thibos)

For a vector-based analysis of defocus, manifest refraction was decomposed into power-vector components (*M, J*_0_*, J*_45_) as defined by Thibos et al. [11]: the SEQ M (= SPH + ½ CYL), the orthogonal astigmatic component *J*_0_ (= −½ CYL cos 2*α*) and the oblique astigmatic component J_45_ ( = −½ CYL sin 2*α*). The vector-based magnitude (DEQ_vec_) was calculated as the Euclidean norm (RMS) of these components:

$${RMS}=\sqrt{{M}^{2}+{{J}_{0}}^{2}+{{J}_{45}}^{2}}$$

This definition corresponds to the distance of the refractive state from emmetropia in the 3-dimensional power-vector space and is identical to the ‘blur strength’ *B* defined by Thibos et al. [11]. An overview of all defocus metrics is provided in Table 1.

**Table 1:** Overview of defocus metrics used in this study.

Metric	Definition	Formula	Interpretation	Reference
SEQ	Spherical equivalent	*SEQ* = *SPH* + ½ *CYL*	Scalar; locates the circle of least confusion relative to the retina	Standard clinical metric
DEQ_abs_	Absolute defocus equivalent	*DEQ*_*abs*_ = |*SPH* + ½ *CYL*| + |½ *CYL*|	Scalar; total dioptric blur combining spherical and cylindrical components	Holladay et al. [9]
DEQ_vec_	Vector-based defocus equivalent (blur strength)	*DEQ*_*vec*_ = *√*(*M*² + *J*₀² + *J*₄₅²)	Vectorial; Euclidean distance from emmetropia in power-vector space; identical to blur strength *B*	Thibos et al. [11]

*CYL* cylinder, *DEQ*_*abs*_ absolute defocus equivalent, *DEQ*_*vec*_ vector-based defocus equivalent, *M* spherical equivalent power-vector component, *SEQ* spherical equivalent, *SPH* sphere, *J*_*0*_*and J*_*45*_ orthogonal astigmatic power-vector components.

For all eyes, distance VAL served as the dependent variable. VAL was defined as the difference between the measured UDVA and the best-CDVA, calculated as:

$${VAL}={UDVA}-{CDVA}$$

The primary predictor was DEQ_abs_, while DEQ_vec_ was evaluated as an alternative metric. By relating VAL to DEQ_abs_ and DEQ_vec_ separately within the defined astigmatic subgroups, the study aimed to determine whether VAL per unit of defocus differs between simple, compound and mixed astigmatism, and whether a vector-based defocus measure offers superior predictive accuracy in specific astigmatic configurations. Analyses were also stratified by IOL type (toric versus non-toric) to assess potential differences related to IOL design.

In addition, *R*² values for the correlation models were compared qualitatively with those obtained in our previously published cohort on DEQs in a broader pseudophakic population, to assess whether restricting the analysis to moderately astigmatic refractions and stratifying by astigmatism type would favour DEQ_vec_ over DEQ_abs_.

### Statistical Analysis

All data were managed using Microsoft Excel (Microsoft, microsoft.com). Statistical analysis was performed using R (Version 4.5.2; R Core Team, r-project.org) via the RStudio interface (Version 2025.09.2+418; Posit, posit.co).

Continuous variables were tested for normality using the Shapiro–Wilk test. Normally distributed data were reported with mean ± standard deviation; non-normally distributed data are described with median and interquartile range. Group comparisons (e.g., between astigmatism types) were performed using analysis of variance (ANOVA). This test is not strictly bound to normally distributed data [14]. For multiple pairwise comparisons, a Bonferroni correction was considered. For non-normally distributed data, non-parametric tests (Wilcoxon–Mann–Whitney) were used [15].

The relationship between VAL and the defocus metrics (DEQ_abs_, DEQ_vec_) was analysed using linear regression through the origin (zero-intercept model). This approach assumes a direct proportionality between defocus magnitude and VAL, as supported by our previous findings in a pseudophakic cohort [10]. For each model, the slope parameter *β* and the coefficient of determination *R*² were calculated. Because the distributions of DEQ_abs_ and DEQ_vec_ do not strictly follow a normal distribution, non-parametric patient-level cluster bootstrapping with 5000 resamples was used to obtain bias-corrected and accelerated (BCa) 95% CIs for the regression slopes [16]. To account for the inclusion of both eyes from 15 patients [17], resampling was performed at the patient level, so that both eyes of a given patient were either included or excluded in each bootstrap sample. As a sensitivity check, analyses were repeated using one randomly selected eye per patient.

Comparisons between astigmatism types and between DEQ_abs_ and DEQ_vec_ were based on descriptive inspection of the overlap of these CIs and on the magnitude of *R*² [18]; no formal statistical test of differences between slopes was performed. Two-sided tests were used for group comparisons, and a *p* value < 0.05 was considered statistically significant.

### Ethics Approval Statement

This study was conducted in accordance with the tenets of the Declaration of Helsinki. Ethical approval was waived by the local Ethics Committee of the Medical Association of Saarland (Ärztekammer des Saarlandes) due to the retrospective nature of the study and the use of de-identified data (approval No. 157/21).

## Results

### Study Population

A total of 75 pseudophakic eyes of 60 patients met the inclusion criteria and were included in the final analysis. The cohort consisted of 39 eyes implanted with a toric IOL and 36 eyes with a non-toric IOL. Both IOL models share an identical aberration-neutral optic design and differ only in the presence of a bitoric component.

Mean UDVA was 0.09 ± 0.16 logMAR, and CDVA was −0.05 ± 0.07 logMAR. The resulting VAL was 0.15 ± 0.14 logMAR. The absolute postoperative cylinder was 0.74 ± 0.24 D, and the SEQ was −0.15 ± 0.62 D (Table 2).

**Table 2:** Descriptive and calculated data.

Parameter	Value (mean ± SD, range)
Number of eyes	75
Toric IOL	39 (52%)
Non-toric IOL	36 (48%)
UDVA (logMAR)	0.09 ± 0.16 (−0.10 to 0.70)
CDVA (logMAR)	−0.05 ± 0.07 (−0.20 to 0.10)
VAL UDVA − CDVA (logMAR)	0.15 ± 0.14 (0.00 to 0.74)
Manifest cylinder (D)	0.74 D ± 0.24 (0.50 to 1.25 D)
Spherical equivalent SEQ (D)	−0.15 D ± 0.62 (−1.75 to +1.25 D)
Absolute defocus equivalent DEQ_abs_ (D)	0.85 D ± 0.41 (0.50 to 2.25 D)
Vector-based defocus equivalent DEQ_vec_ (D)	0.66 D ± 0.34 (0.35 to 1.82 D)

The number of eyes refers to the number of included datasets in this project. *CDVA* best corrected distance visual acuity. *DEQ*_*abs*_ defocus equivalent, *IOL* intraocular lens, *UDVA* uncorrected distance visual acuity, Visual Acuity Loss (VAL) = UDVA − CDVA (logMAR). The manifest cylinder is based on subjective, distance-corrected refraction. *SEQ* spherical equivalent, *DEQ*_*vec*_ vector-based version of the DEQ, *D* dioptre, *SD* standard deviation.

### Global Relation Between Visual Acuity Loss and Defocus

**Fig. 1:**
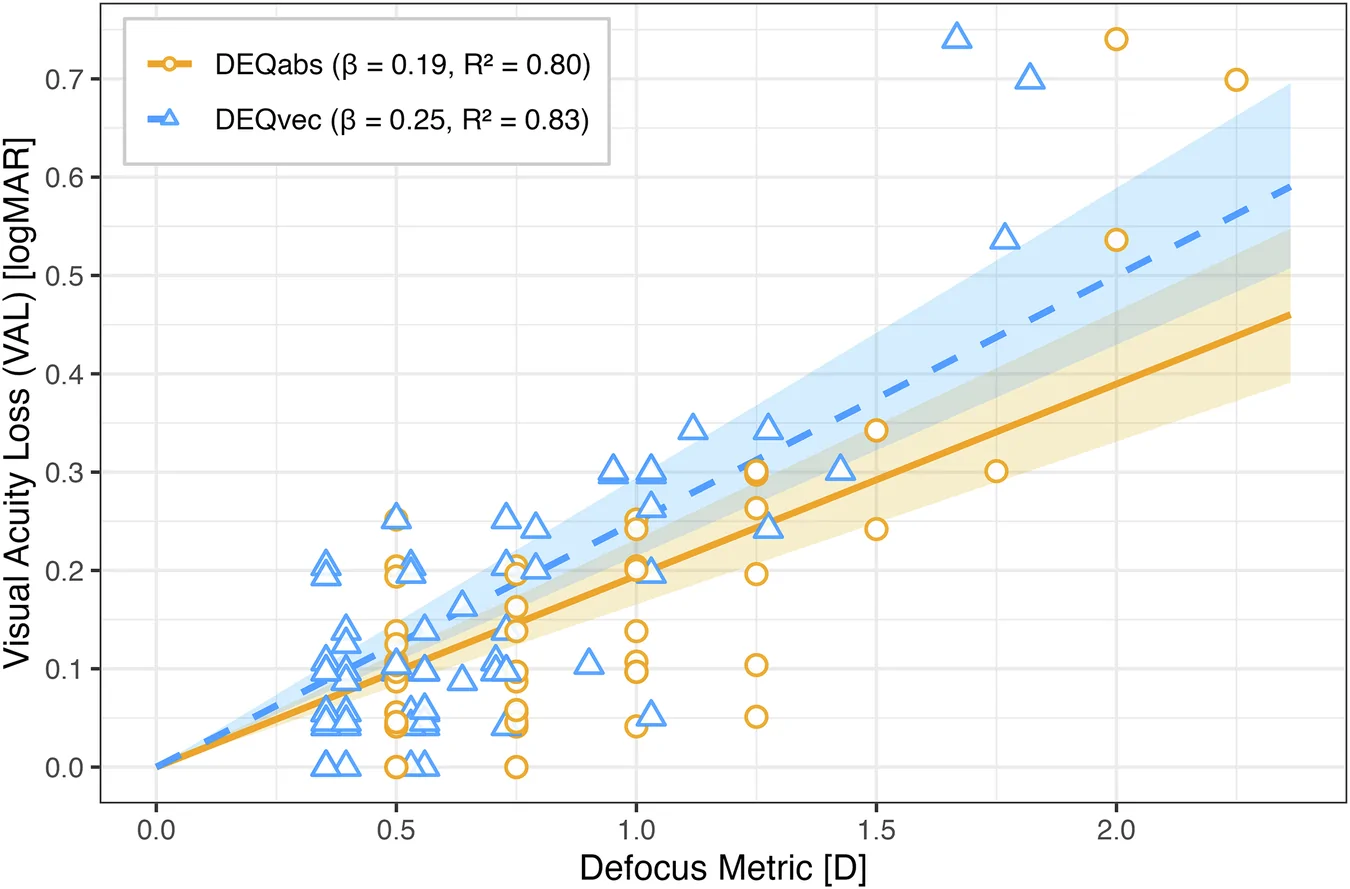
Visual acuity loss (VAL; uncorrected distance visual acuity [UDVA] minus best corrected distance visual acuity [CDVA], logMAR) as a function of absolute defocus equivalent (DEQ_abs_) and vector-based defocus equivalent (DEQ_vec_) in 75 pseudophakic eyes with residual cylinder between 0.50 and 1.25 D. Solid lines show proportional regression fits constrained to pass through the origin with 95% bias-corrected and accelerated (BCa) confidence bands (shaded). In both cases, the relationship is almost linear; DEQ_vec_ yields a slightly steeper slope and marginally higher coefficient of determination (*R*^2^) than DEQ_abs_. D, dioptre.

Across all 75 eyes, VAL increased approximately linearly with DEQ_abs_. Linear regression through the origin yielded a slope of *β* = 0.195 logMAR per dioptre (bootstrap BCa 95% CI: 0.165 to 0.232; *R*^2^ = 0.80) (Fig. 1). For the DEQ_vec_, the analysis produced a slightly steeper slope of *β* = 0.250 logMAR per dioptre (BCa 95% CI: 0.215–0.294) and a higher *R*² (0.83). Thus, in this cohort of moderately astigmatic pseudophakic eyes, both DEQ_abs_ and DEQ_vec_ were strong predictors of VAL.

### Distribution of Astigmatism Types (Sturm’s Conoid)

Based on the position of the two principal focal powers relative to the retinal plane, 29 eyes (39%) were classified as having simple myopic astigmatism and 21 (28%) as having simple hyperopic astigmatism. Twelve eyes (16%) showed compound myopic astigmatism, six eyes (8%) compound hyperopic astigmatism and seven eyes (10%) mixed astigmatism. For the main analysis, simple myopic and simple hyperopic cases were pooled into a simple group (*n* = 50), while compound myopic and compound hyperopic cases were pooled into a compound group (*n* = 18). Mixed astigmatism (*n* = 7) was analysed separately.

### Pupil Size Analysis

To assess whether pupil size acted as a confounding factor (pinhole effect), the pupil diameter was analysed across the different astigmatism types. Mean pupil size in the overall cohort was 3.50 ± 0.82 mm (range: 2.0–5.6 mm). There was no statistically significant difference in pupil diameter between the groups (simple: 3.60 ± 0.82 mm; mixed: 3.14 ± 0.68 mm; compound: 3.37 ± 0.88 mm; ANOVA *p* = 0.29). This suggests that the shallower VAL observed in simple astigmatism is unlikely to be driven by an increased depth of focus from miosis.

### Influence of Astigmatism Type on the DEQ–VA Relationship

**Fig. 2:**
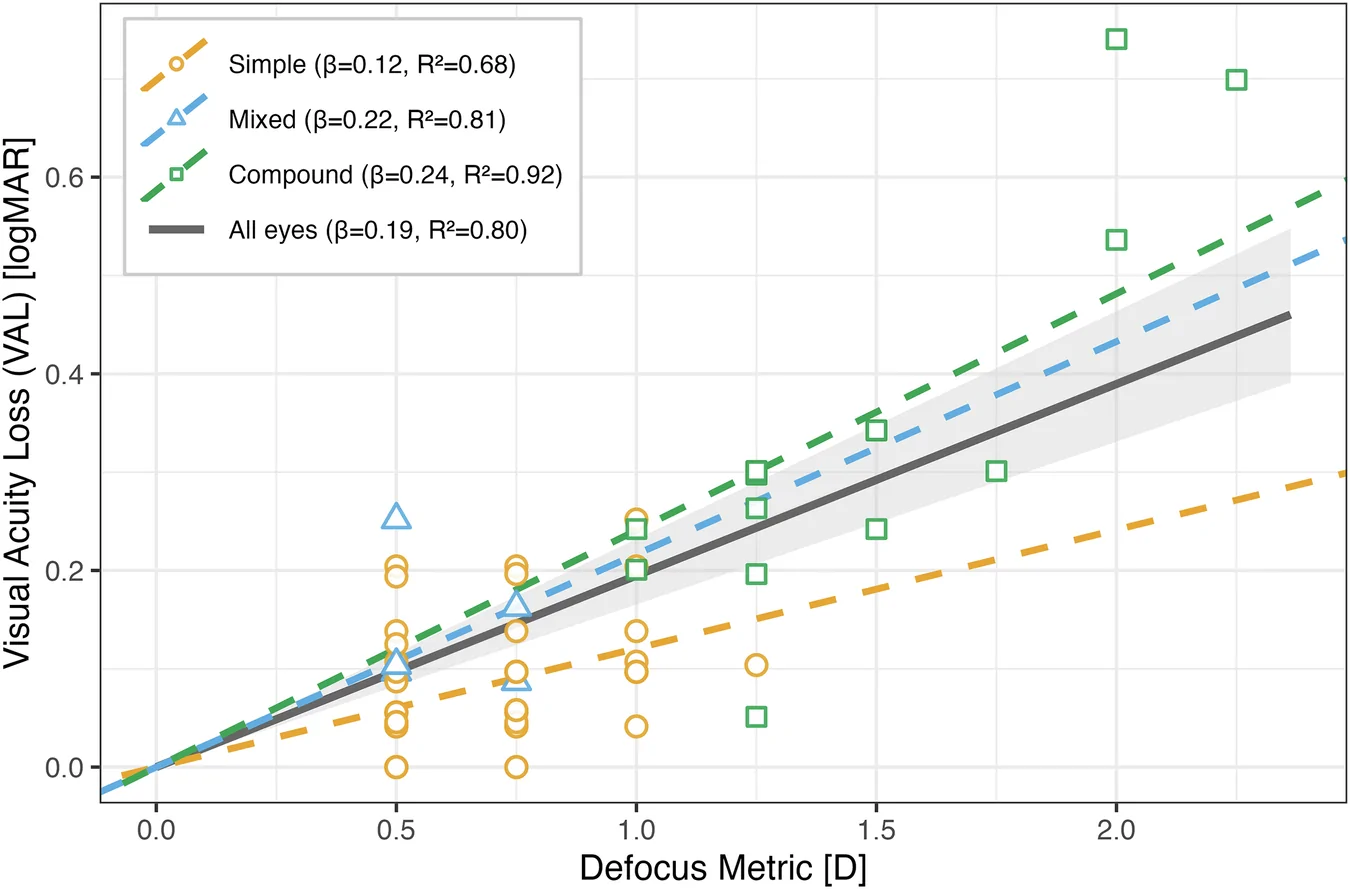
Visual acuity loss (VAL; uncorrected distance visual acuity [UDVA] minus best corrected distance visual acuity [CDVA], logMAR) as a function of absolute defocus equivalent (DEQ_abs_), stratified by the type of astigmatism. Symbols represent individual eyes classified as simple, mixed or compound astigmatism. Dashed lines show proportional regression fits for each subgroup, and the solid line represents the fit for all eyes with 95% bias-corrected and accelerated (BCa) confidence band (shaded). Simple astigmatism exhibits a noticeably flatter slope than compound astigmatism, with mixed astigmatism showing an intermediate behaviour. D, dioptre.

When eyes were stratified by the type of astigmatism, the slope of VAL versus DEQ differed markedly between groups (Table 3, Fig. 2). With DEQ_abs_ as the predictor, the proportional regression slope was approximately 0.12 logMAR per dioptre in simple astigmatism, 0.22 logMAR per dioptre in mixed astigmatism and 0.24 logMAR per dioptre in compound astigmatism (0.195 logMAR/D in the overall cohort). Consequently, simple astigmatism demonstrated a notably flatter relationship, exhibiting approximately half the VAL per dioptre of DEQ_abs_ compared with the compound group, whereas mixed astigmatism showed an intermediate behaviour. Despite the shallower slope, the model fit in the simple group remained robust (*R*² ≈ 0.68), while in compound configurations *R*^2^ exceeded 0.90.

**Table 3:** Linear regression of visual acuity loss (VAL) (UDVA − CDVA, logMAR) on scalar and vector-based defocus equivalents (DEQ_abs_, DEQ_vec_) in the whole cohort and stratified by the type of astigmatism.

Group	*n*	*β* DEQ_abs_ (logMAR/D)	BCa 95% CI DEQ_abs_	*R*² DEQ_abs_	*β* DEQ_vec_ (logMAR/D)	BCa 95% CI DEQ_vec_	*R*² DEQ_vec_
All eyes	75	0.195	0.165–0.232	0.80	0.250	0.215–0.294	0.83
Simple	50	0.121	0.099–0.146	0.68	0.165	0.135–0.200	0.68
Simple myopic	29	0.142	0.107–0.179	0.70	0.194	0.149–0.244	0.69
Simple hyperopic	21	0.092	0.072–0.113	0.76	0.127	0.099–0.155	0.77
Mixed	7	0.216	0.161–0.330	0.81	0.236	0.183–0.344	0.83
Compound	18	0.241	0.203–0.284	0.92	0.294	0.250–0.347	0.92

Slope *β* is given in logMAR per dioptre of DEQ, with bias-corrected and accelerated (BCa); 95% confidence intervals (CIs) based on non-parametric patient-level cluster bootstrapping. *R*² was calculated for models constrained to pass through the origin.

*CDVA* best corrected distance visual acuity, *D* dioptre, *DEQ*_*abs*_ defocus equivalent, *DEQ*_*vec*_ vector-based version of the DEQ, *UDVA* uncorrected distance visual acuity.

When the simple astigmatism group was stratified by the sign of the spherical error, simple myopic eyes (*n* = 29) showed a steeper slope (*β* = 0.142 logMAR/D, BCa 95% CI: 0.107–0.179, *R*² = 0.70) than simple hyperopic eyes (*n* = 21; *β* = 0.092 logMAR/D, BCa 95% CI: 0.072–0.113, *R*² = 0.76). Both subgroups remained well below the compound group (*β* = 0.241), confirming that the lower VAL in simple astigmatism is consistent across both subtypes.

The same qualitative pattern was observed when DEQ_vec_ was used as the predictor (Table 3). Slopes were slightly steeper and *R*^2^ marginally higher than for DEQ_abs_ in all three groups, but the relative ranking of astigmatism types remained unchanged: compound astigmatism showed the steepest slope and highest *R*^2^, mixed astigmatism an intermediate slope and simple astigmatism the lowest slope with the least VAL per dioptre of defocus.

### Influence of IOL Type on the DEQ–VAL Relationship

Stratification by IOL type yielded similar slopes for toric (*β* = 0.185 logMAR/D, BCa 95% CI: 0.139–0.221, *R*² = 0.80) and non-toric eyes (*β* = 0.210 logMAR/D, BCa 95% CI: 0.159–0.260, *R*² = 0.81), with broadly overlapping CIs. Both IOL types showed comparable *R*^2^, indicating that the relationship between DEQ and VAL was equally well described regardless of whether the residual astigmatism originated from incomplete toric correction or from uncorrected corneal astigmatism.

### Inter-eye Correlation and Sensitivity Analysis

Patient-level cluster bootstrapping yielded CIs virtually identical to those obtained by eye-level resampling (CI width changes <2% across all groups), confirming that intra-subject correlation had a negligible impact on the estimates. A sensitivity analysis using one randomly selected eye per patient produced stable slope estimates and confirmed the robustness of the findings.

## Discussion

This study analysed how the types of residual astigmatism, described by the position of Sturm’s conoid, modulate VAL in pseudophakic eyes with a moderate amount of residual cylinder. Across the entire cohort, VAL increased nearly proportionally with DEQ_abs_, with a slope of 0.195 logMAR per dioptre and an *R*^2^ of 0.80. This confirms and extends our previous finding in a broader pseudophakic population, where a similar proportionality between DEQ and VAL was observed. The data show that this relationship remains valid when the analysis is restricted to eyes with clinically relevant residual astigmatism after implantation of modern aspheric IOLs.

### Influence of Astigmatism Type

A key finding of this study is that the slope of the DEQ–VAL relationship is not constant across all astigmatic types. When eyes were stratified according to the position of the two principal focal lines relative to the retina, systematic differences were observed: Simple astigmatism showed the lowest slope (approximately 0.12 logMAR per dioptre DEQ_abs_), whereas compound astigmatism exhibited a much steeper slope (0.24 logMAR per dioptre). Mixed astigmatism fell between these extremes with a slope of 0.22 logMAR per dioptre. In other words, for the same increase in DEQ, compound configurations resulted in approximately twice as much VAL as simple astigmatism.

These differences are optically plausible. In simple astigmatism, one principal focal line is located on or close to the retinal plane, while the other lies in front of or behind the retina. Consequently, the retinal image still contains a relatively well-focused component in one meridian, which may support letter recognition and mitigate the functional impact of blur. The magnitude of this effect will depend on optotype orientation relative to the blurred meridian. In contrast, in compound astigmatism, both focal lines are located in front of or behind the retina; the entire image is defocused, and the resulting blur cannot be compensated by a ‘sharp’ meridian. Mixed astigmatism represents an intermediate scenario, with one focal line in front of and the other behind the retina, positioning the COLC near the retinal plane. The present results demonstrate that these optically distinct configurations are not equivalent, even when the scalar amount of blur summarised by DEQ_abs_ is identical.

*R*^2^ values support this interpretation. In compound astigmatism, *R*^2^ exceeded 0.90, indicating a strong association between residual defocus and uncorrected acuity. In simple astigmatism, the fit was less robust (*R*² ~ 0.68) and the slope lower, suggesting that additional factors such as letter orientation, neural adaptation or minor refractive variations may play a larger role.

Nonetheless, the ranking of slopes was consistent across all models and remained stable when a vector-based defocus metric was applied. Within the simple astigmatism group, simple myopic eyes showed a steeper slope than simple hyperopic eyes (*β* = 0.142 vs. 0.092 logMAR/D). Although both subtypes remained well below compound astigmatism, this difference is consistent with the hypothesis that positive corneal spherical aberration may partially mitigate hyperopic defocus. However, both subgroups were relatively small, and the CIs overlapped, so this observation should be regarded as exploratory.

Similarly, the mixed astigmatism group contained only seven eyes, which limits the precision of the slope estimate in this category. While the intermediate position of mixed astigmatism between simple and compound is optically plausible, the exact slope should be interpreted with caution and requires confirmation in larger cohorts.

### Scalar Versus Vector-Based Defocus Equivalent

The vector-based defocus equivalent, DEQ_vec_, yielded slightly steeper slopes and marginally higher *R*^2^ than DEQ_abs_ across all groups. This finding aligns with optical theory, as DEQ_vec_ represents the Euclidean norm of the refractive error within the three-dimensional power-vector space (*M, J*_0_*, J*_45_), thereby retaining more information regarding the direction of the refractive error. In a sample dominated by astigmatic refractions, such a metric can capture subtle differences that are lost when absolute scalar values are merely summed. However, the improvement in *R*^2^ was modest and, importantly, DEQ_vec_ did not alter the relative ranking of the astigmatism types. From a clinical perspective, this suggests that DEQ_abs_ remains a practical scalar measure for describing the magnitude of residual blur, whereas DEQ_vec_ may be useful when more detailed modelling is required or when comparing different refraction patterns in research settings.

### Clinical Implications for IOL Power Targets

The clinical relevance of these findings becomes apparent when considering how IOL power is typically selected in everyday practice. Modern IOL calculation formulas, as implemented in common biometers, report the predicted postoperative refraction as a target SEQ. In an astigmatic eye in which no toric IOL is implanted, aiming for an emmetropic SEQ (0.00 D) typically results in a mixed astigmatism configuration. In this state, one principal focus line lies in front of the retina and the other one behind it, placing the COLC on or near the retinal plane.

While this configuration appears balanced in terms of defocus and is often implicitly considered as ‘neutral’, the current data suggest that it may not be the optimal strategy for minimising VAL. Within the cylinder range of 0.50–1.25 D, a simple myopic type exhibited approximately half the VAL per dioptre of DEQ_abs_ of compound astigmatism and substantially less VAL than mixed astigmatism.

Therefore, from a functional standpoint, it may be advantageous in selected cases to accept a slight myopic bias resulting in simple myopic astigmatism. In this configuration, one focal line is located on the retina while the second lies anterior to it. For patients who tolerate mild myopia and prioritise UDVA, such a strategy could attenuate the functional impact of residual astigmatism without altering the magnitude of the cylinder. Because the present data are observational and retrospective, this clinical implication should be regarded as hypothesis-generating and would need to be confirmed in a prospective interventional study before being adopted in routine practice.

The present results do not imply that surgeons should abandon the goal of an emmetropic SEQ. Rather, they highlight that in non-toric eyes with relevant corneal astigmatism, the choice of target refraction implicitly selects a particular configuration of Sturm’s conoid and that different configurations with the identical DEQ are not functionally equivalent. Incorporating knowledge of the DEQ–VAL relationship for simple, mixed and compound astigmatism into IOL planning and patient counselling may help to tailor postoperative refraction better to individual visual needs.

### IOL Type

Stratification by IOL type (toric versus non-toric aspheric lenses of the same platform) showed very similar slopes and broadly overlapping CIs. Both IOL models share an identical aberration-neutral optic design, differing only in the presence of a bitoric component, so that any residual astigmatism is captured by the DEQ regardless of its origin. This supports the decision to pool all eyes for the main analysis.

### Limitations

However, this study had some limitations. First, the analysis was retrospective and conducted at a single centre, which may limit generalisation of the results. The distribution of astigmatism types was not controlled but resulted from routine clinical practice; in particular, the mixed astigmatism group comprised only seven eyes and the compound hyperopic subgroup six eyes. As a consequence, CIs in these categories are wider, and the slope estimates for these subgroups should be interpreted with caution.

Second, only eyes with a manifest cylinder between 0.50 and 1.25 D were included. This range was chosen to represent clinically relevant residual astigmatism in contemporary cataract surgery and to avoid extreme outliers. The findings may not apply directly to very low (<0.50 D) or high (>1.25 D) astigmatism or to eyes with irregular corneal astigmatism. In cases of minimal astigmatism, the distinct influence of the type of astigmatism likely diminishes as the resulting blur circle remains within the physiological depth of focus. Conversely, for higher astigmatism, it is anticipated that the relative advantage of simple astigmatism, driven by the preservation of one focused meridian, would persist, although the absolute visual degradation would be more profound across all groups.

Third, the analysis addressed only distance visual acuity; intermediate and near visual function, contrast sensitivity and subjective quality of vision were not assessed systematically. It is conceivable that the relative advantages of different astigmatic configurations vary with viewing distance and illumination.

Fourth, residual refraction was characterised solely by low-order spherocylindrical components. Higher-order aberrations, intraocular scattering and neural factors were not measured and may have contributed to the unexplained variance, particularly in simple astigmatism, where the DEQ–VAL relationship was flatter. However, pupil size, a major determinant of depth of focus and optical image quality, showed no significant difference between the astigmatism groups (*p* = 0.29). This indicates that the observed visual acuity benefit in simple astigmatism is not driven by pupil-dependent optical factors, but rather by the configuration of the spherocylindrical error itself.

Finally, a simplified classification of astigmatism types was used, based on principal powers with a tolerance band around the retinal plane. Although this reflects standard textbook definitions, small variations in the thresholds could shift individual cases between the astigmatic categories.

Additionally, the orientation of the cylinder axis was not considered in this analysis. The relationship between axis orientation and visual acuity depends on optotype design, and the uneven distribution of axis orientations in the present sample (against-the-rule *n* = 51, oblique *n* = 14, with-the-rule *n* = 10) did not permit a meaningful stratification.

Lastly, the comparison of regression slopes between astigmatism types was based on the overlap of bootstrap-derived CIs and should be regarded as descriptive rather than as a formal statistical test of differences between slopes. A formal inferential comparison of slopes was not performed, and the numerical ranking across simple, mixed and compound astigmatism therefore awaits confirmation in larger, prospectively designed cohorts.

## Conclusion

The present study provides evidence that the functional impact of residual astigmatism in pseudophakic eyes depends not only on its magnitude but also on the configuration of Sturm’s conoid, specifically the type of astigmatism. For the same DEQ, compound astigmatism produces the steepest and most predictable VAL, mixed astigmatism an intermediate VAL and simple astigmatism—particularly simple myopic astigmatism—the lowest slope. Vector-based DEQ may slightly improve model fit, but does not change this ranking.

These findings support the use of DEQ as a practical descriptor of residual blur and add the important nuance that the type of astigmatism modulates its functional consequences. Future work should explore how these insights can be integrated into IOL power calculation strategies and whether deliberate targeting of favourable astigmatic types, such as mild simple myopic astigmatism, can be used prospectively to optimise uncorrected visual acuity while potentially supporting pseudo-accommodation in patients who do not receive toric IOLs.

## Data Availability

The data that support the findings of this study are available from the corresponding author upon reasonable request.
